# Developing a complex vocational rehabilitation intervention for patients with inflammatory arthritis: the WORK-ON study

**DOI:** 10.1186/s12913-023-09780-2

**Published:** 2023-07-08

**Authors:** Christina Merete Tvede Madsen, Jette Primdahl, Ann Bremander, Linda Eggen, Jeanette Reffstrup Christensen

**Affiliations:** 1 Danish Hospital for Rheumatic Diseases, University Hospital of Southern Denmark, Engelshøjgade 9A, 6400, Sønderborg, Denmark; 2 Department of Regional Health Research, University of Southern Denmark, Odense, Denmark; 3 Sygehus Sønderjylland, University Hospital of Southern Denmark, Aabenraa, Denmark; 4 Section of Rheumatology, Department of Clinical Sciences Lund, Lund University, Lund, Sweden; 5 Department of Public Health, Research Unit of General Practice, University of Southern Denmark, Odense, Denmark; 6 Department of Public Health, User Perspectives and Community-Based Interventions, University of Southern Denmark, Odense, Denmark; 7 Research Unit of General Practice, Aarhus University, Aarhus, Denmark

**Keywords:** Complex intervention, Development process, Work ability, Axial spondylarthritis, Rheumatoid arthritis, Psoriatic arthritis

## Abstract

**Background:**

People with inflammatory arthritis often experience challenges at work and balancing paid work and energy in everyday life. Low work ability is common, and people with inflammatory arthritis face high risks of losing their jobs and permanent exclusion from the labour market. Context-specific tailored rehabilitation targeting persons with inflammatory arthritis is limited. The aim of this study is to describe the development of WORK-ON – a vocational rehabilitation for people with inflammatory arthritis*.*

**Methods:**

Following the Medical Research Council’s framework for complex interventions, WORK-ON was developed based on existing evidence, interviews with patients and rehabilitation clinicians, a workshop, and an iterative process.

**Results:**

The six-month vocational rehabilitation, WORK-ON, consists of 1) an initial assessment and goal setting by an occupational therapist experienced in rheumatology rehabilitation, 2) coordination by the same occupational therapist and individual support, including navigating across the primary and secondary health sectors, as well as social care, 3) group sessions for peer support, and 4) optionally individually tailored consultations with physiotherapists, nurses, or social workers.

**Conclusion:**

WORK-ON is ready to be tested in a feasibility study.

**Trial registration:**

The Regional Committees on Health Ethics for Southern Denmark stated that no formal ethical approval was necessary in this study (20,192,000–105).

**Supplementary Information:**

The online version contains supplementary material available at 10.1186/s12913-023-09780-2.

## Introduction

Inflammatory arthritis (IA), (IA encompasses rheumatoid arthritis (RA), axial spondyloarthritis (axSpA), and psoriatic arthritis (PsA)) are chronic inflammatory diseases characterised by swelling of the joints, stiffness, fatigue, pain, mental distress, and reduced mobility [1–4]. In Denmark, about 50,000 people have been diagnosed with RA, and more than 30,000 have been diagnosed with PsA or axSpA [5]. Even though pharmacological and surgical treatments have improved, people with IA still experience physical disabilities and psychosocial challenges [6–9].

People with IA often experience challenges at work, how to maintain their job and find it especially challenging to balance paid work and energy in everyday life [10–12]. Low work ability and increased risks of sick leave and long-term-sickness absenteeism are common among people with IA [13–18]. Continuing normal everyday occupations including work is one of the most important elements when trying to maintain the everyday life people with IA had before their IA diagnosis [7]. They often try to prioritise energy for work at the expense of social occupations and leisure activities [10–12, 19].

In addition, people with IA face high risks of losing their jobs and permanent exclusion from the labour market, and up to 40% lose their jobs in the first years after being diagnosed with IA [13–18, 20]. Because of the reduced capacity to work, IA has economic consequences for the individual as well as for society [9, 13]. In addition, participation in paid work is central for the individuals’ identity, sense of belonging, and social roles [9, 11]. Furthermore, work contributes to good health, well-being, quality of life, and recovery for people with chronic health conditions [6, 9–12, 21]. In a systematic review on job loss prevention interventions targeted towards people with IA, we found that some strategies may have an effect on work ability, absenteeism, and job loss [22]. The review included six studies with inconsistent results due to the heterogeneity in the different interventions and the outcome measures. The interventions were also sparsely described, which made them difficult to replicate. Vocational rehabilitation (VR) depends on the context, and most countries have different social security systems. The systematic review pointed to a need for developing a context specific tailored rehabilitation. We therefore developed a VR adjusted to the Danish context inspired by VRs that had showed positive results. In the Danish context it needed to secure coherence between the hospitals, the municipal job centres, and the workplaces [22].

The aim of this study was to describe the process of developing a context-specific evidence – and theoretically based VR called WORK-ON.

## Methods

### Study design

**Fig. 1:**
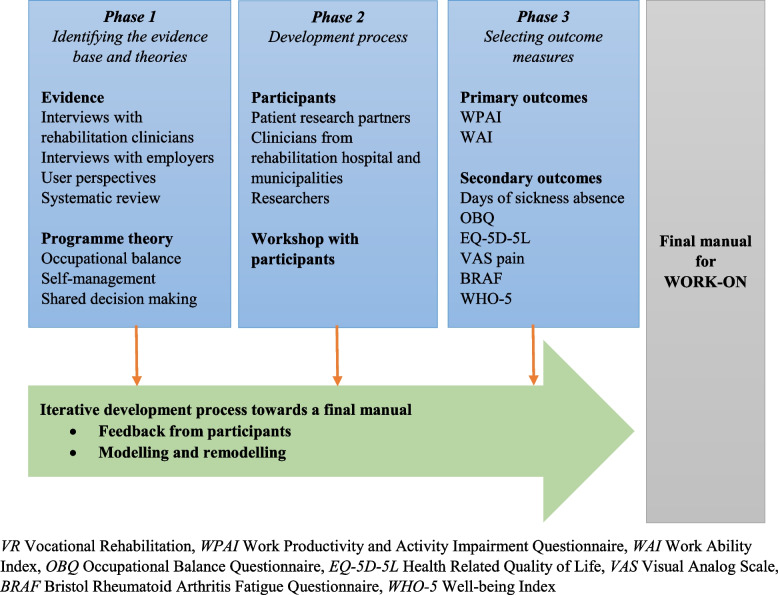
Development of the VR

The development process was based on the Medical Research Council’s (MRC) updated framework for complex interventions [23], as multiple components and different health professionals are needed in VR. The MRC framework describes a systematic way to develop, feasibility test, implement, and evaluate complex interventions [23]. MRC emphasises that initially, the evidence base and relevant theory should be identified. Secondly, the intervention should be modelled and relevant outcomes selected. Thirdly, a feasibility test of the intervention should be performed. Finally, the effectiveness of the intervention should be evaluated in a larger randomised controlled trial (RCT) [23]. The present paper describes the development of a VR up to the point at which it is ready to be tested in a feasibility study. The Template for Intervention Description and Replication (TIDieR) was used to report the phases of the development process (please see Supplementary File 1) [24].The VR was developed in three phases between May 2020 and March 2022 following the MRC framework (please see Fig. 1).

### Programme development

#### Phase 1: Identifying the evidence base and theory models

The evidence base consisted of our previous systematic review [22] and a qualitative study on user perspectives on perceived challenges at work and need for support among people with IA [10]. We found that people with IA described the need for recognition and understanding from their employers, colleagues, and family to be able to keep their jobs [10]. Flexibility in every possible way at work was essential for them to remain at work, and positive cooperation and communication between the employer, the employee, and the job centre in the municipality is important. Further, meeting with others who are in the same situation was emphasised [10, 25, 26].

In addition, individual interviews with 21 multidisciplinary RCs from hospital and municipalities were conducted to explore their views. The interview guide included questions such as: ‘Which challenges do you experience that people with IA face in their work?’; ‘What are your best experiences with VR’; ‘Do you involve the employer, co-workers, and relatives?’; and ‘Which type of professional support do you experience as the most important in VR to patients with IA?’ (please see Supplementary file 2). The participants mentioned group sessions, peer support, coordination, energy management, involvement of employers and relatives, individual support, and individual consultations with occupational therapists (OTs), physiotherapists, nurses, and social workers.

Additionally, 13 interviews were conducted with employers to explore their perspectives of job loss prevention for persons with IA. The interview guide included questions such as: ‘What are your experiences with job retention and employees with IA?’; ‘How will you describe your cooperation with the municipal job centre?’; ‘What type of information do you need in relation to prevent job loss at an employee with IA?’’ and ‘Do you experience that the employee has difficulties with balancing work and everyday life?’ (please see Supplementary file 3). The employers were concerned about politics regarding sick leave as they experienced it as complicated and time consuming. Furthermore, the relations between employee and employer, participation in work, and cooperation with the job centre were issues of importance to the employers. These results highlighted the importance of involving the employers in VR [27].

The results of interviews with rehabilitation clinicians (RC) and employers will be published in separate papers.

When developing the present programme, we included occupational balance [28], self-management [29], self-efficacy [30], and shared decision making [31] as key theories (please see Fig. 1). Finding a balance between paid work and energy in everyday life was especially important for people with IA [10]. In general, occupational balance describes an individual’s satisfaction with occupations in life, the variations between them, and how meaningful they are [28, 32]. Occupational balance is also characterised as the experience of having the right number of occupations to balance time use. In this context, occupational balance involves areas such as paid work, self-care, leisure, and sleep, as well as occupations with different characteristics, including obligatory, voluntary, and paid work [28, 32, 33].

Self-management is defined as the ability to manage symptoms, treatments, physical and psychosocial consequences from living with a chronic health condition [29]. To be able to manage a chronic health condition, patients have to manage their cognitive, behavioural, and emotional reactions to maintain a satisfactory quality of life. Health professionals must support the patients in making their own decisions, solving their own problems, seeing their own potential, controlling their own situations, and being active in their daily lives [29, 31, 34]. Self-management is often underpinned by the concept of self-efficacy. The self-efficacy concept relates to beliefs about one’s perceived abilities or inabilities to complete a specific task, and not to one’s actual capabilities or performance [30]. This reflects the individual’s subjective assessment of their abilities and skills to successfully achieve their goals [35].

When patients are learning how to manage their own situations, it is relevant to look at how goal setting and decision making are performed in cooperation between patients with IA and RCs. Shared decision making is a process and gives opportunities to reflect upon goal setting, wishes, hopes, needs and dreams [31]. It shifts the power and control between the patient and the clinician and makes the interaction equal. Shared decision making is described as a process that consists of three steps: 1) a team talk in which patients and clinicians work together as a team to make decisions regarding care, 2) the option talk, in which opportunities are discussed, and 3) the decision talk to make preference-based decisions [31]. Shared decision making and self-management contribute to ensuring that the patient takes active ownership of his or her process to gain most out of the VR.

**Fig. 2:**
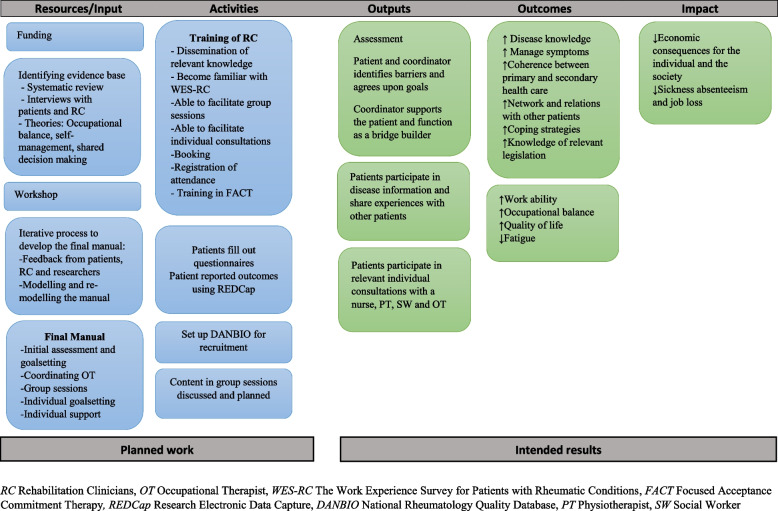
Logic model

The programme theory was depicted in a logic model which illustrates the relationship between the planned work (resources/inputs and activities and the intended results (outputs, outcomes and impact). A logic model provides an overview of how the intervention works and mechanisms of impact [36]. (Please see Fig. 2).

#### Phase 2: Development process

Four groups of participants were involved in the development phase: 1) patient research partners, 2) RCs from the Danish Hospital of Rheumatic Diseases (DHRD), 3) RCs from a municipal job centre in the Region of Southern Denmark, and 4) researchers working in the field of rehabilitation of people with IA.

All results from phase 1 led to a description of the VR (WORK-ON) in a detailed manual. Through an iterative process, the VR was modelled and remodelled several times through continuous feedback from the participants, leading to the development of the final manual. The participants decided the type of feedback that suited them best. Written feedback was given via email, and oral feedback was given online or by phone on the consecutive versions of the manual. The participants reflected on the dose, length, and content of WORK-ON and whether the offer was meaningful to the patients.

When a draft version of the manual was reached, a workshop was held with ten participants. All participants received an email with the draft manual before the workshop. The workshop encompassed a presentation of the participants, a summary of the content in the planned VR, and feedback from the participants on each part of the VR.

The draft manual was translated into English and sent to two VR researchers at the University of Salford, who gave feedback in an online meeting at which outcome measures were also discussed. The manual was further modelled and remodelled several times. As a final step, the manual was read and commented by the RCs who were to deliver the VR. The manual seemed transparent to them, and no further adjustments were made. Thus, the manual and the content was ready for a subsequent feasibility test.

### Inclusion and exclusion criteria

Inclusion and exclusion criteria for patients to be offered WORK-ON were discussed and decided in phase 1.

#### Inclusion criteria

- Aged ≥ 18
- Diagnosed with RA, axSpA, or PsA
- In paid work (full or part time, self-employed, or taking an education)
- Not on long term sick leave (or if on sick leave, this must be less than four weeks)
- Able to read and understand Danish
- Answers *unlikely* or *not certain* to question #6 from the Work Ability Index Questionnaire: *‘Do you believe, according to your present state of health, that you will be able to do your current job two years from now?’* [41].

#### Exclusion criteria

- Not able to attend WORK-ON because of dementia or other cognitive issues
- Is assessed to be in a non-stable phase because of activity in their IA. There is a need for pharmacological adjustment
- Waiting for or has planned alloplastic, operations of joints (hands, knees, hip, etc.) or major surgery within the next six months
- Problems maintaining work is assessed not because of IA but because of other comorbidity such as psychiatric illness, heart disease, or chronic obstructive pulmonary disease

#### Phase 3: Selecting outcome measures

Baseline information (age and gender) and patient reported outcome measures (PROMs) will be collected at baseline and six months later in the feasibility study. The primary outcome is work ability, which will be measured with both the Work Productivity and Activity Impairment questionnaire (WPAI) [42] and the Work Ability Index (WAI) [43]. Both measurements will be included in the feasibility study to be able to evaluate which is the better as a primary outcome in a later RCT. Key secondary outcomes were number of days of sickness absence in relation to IA reported each month through the intervention period by text messages, Occupational Balance measured with the Occupational Balance Questionnaire (OBQ) [44, 45], and health-related quality of life measured with EQ-5D-5L [46]. EQ-5D-5L was chosen to enable health economic analyses in a later RCT. Additional secondary outcomes were pain measured by asking the experience of pain the last four weeks and how much physical pain affected work and household chores, fatigue measured with the Bristol Rheumatoid Arthritis Fatigue questionnaire (BRAF) [47], and well-being by the WHO-5 well-being index [48]. These outcomes were chosen, as it seemed they have an influence on the participants’ work ability [22].

## Results

**Fig. 3:**
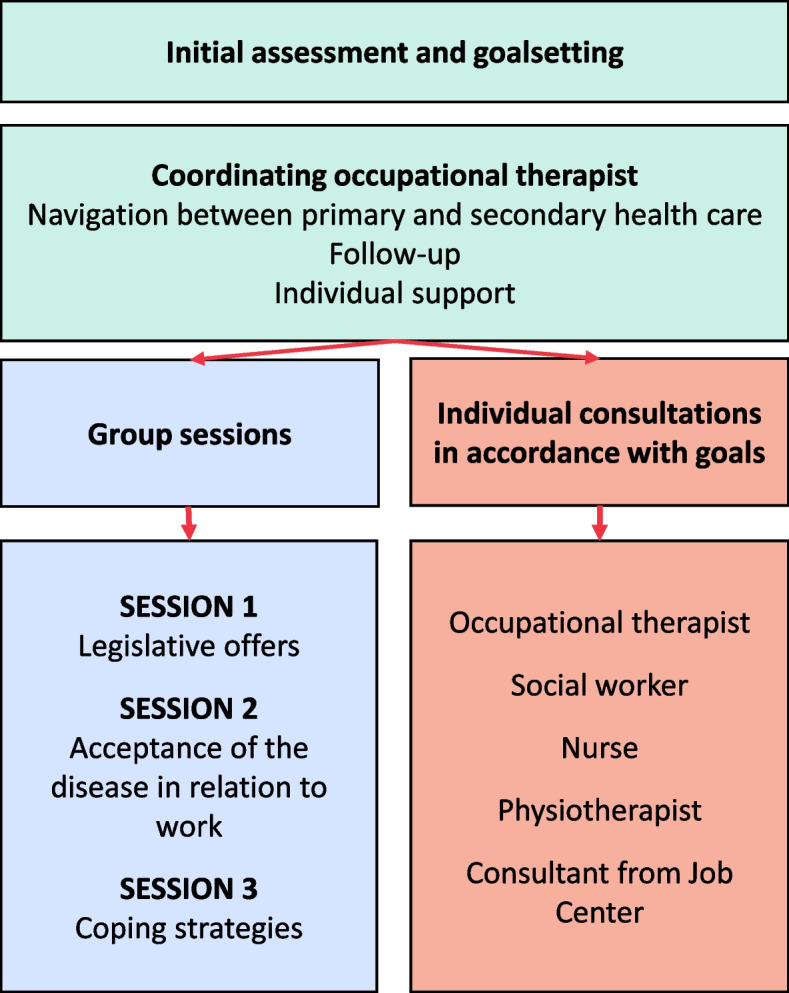
Content of the VR

**Fig. 4:**
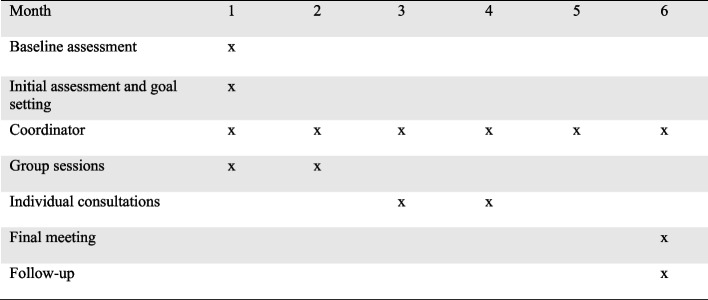
Timeline of the VR

The development resulted in four parts of the intervention which are described in the final manual. The four parts are: 1) initial assessment and goal setting by an OT experienced in rheumatology rehabilitation, 2) coordination and individual support by the same OT throughout the VR, including support in navigating across primary and secondary health and social care, 3) group sessions with presentations and discussions to stimulate peer support, and 4) needs based individually tailored VR consultations with physiotherapists, nurses, or social workers (please see Fig. 3). The duration of WORK-ON was decided to be six months and includes 9 to 18 meetings, depending on the participants’ needs. The first three months will have the highest intervention intensity and the following three months will have a lower intensity and will thus instead make room for reflexion, implementation of new strategies and follow ups. Similar, but not VR interventions in other Danish settings have shown to be feasible with just three months’ duration, which also can be managed long term in clinical practices (please see Fig. 4) [37–39].

Findings from interviews with patients and rehabilitation clinicians illustrated that patients with IA need support with coordination between the primary and secondary health care system to help to keep an overview of their VR process and support coherence [10]. Evidence shows that VR delivered by OTs may have a positive effect on work ability [22]. OTs are trained in energy management, occupational balance, and goal setting. With this in mind, we chose an OT for the coordinating OT role [40]. Being a part of a group and sharing experiences with other patients at risk of losing their jobs could have an effect on work ability and self-efficacy [22, 30].Furthermore, patients with IA often feel alone with their challenges at work and express a need to meet with other peers [25, 26]. Based on these findings, we chose to include three group sessions in WORK-ON.

### The final content in WORK-ON

#### Initial assessment and goal setting

Potential participants will be invited through DANBIO, a national rheumatology quality database [49]. A two-hour physical meeting with an initial assessment and goal setting process is performed by a coordinating OT at the DHRD. The coordinating OT has experience with rehabilitation of patients with IA and the challenges they face in the labour market. RCs at the DHRD have the International Classification of Functioning, Disability and Health (ICF) as their preconception as ICF is a rehabilitation framework [50]. Furthermore, they have a person-centred approach and are trained in Focused Acceptance and Commitment Therapy (FACT) and motivational interviewing [51, 52]. The two-hour initial meeting starts with a structured interview guided by the Work Experience Survey for Patients with Rheumatic Conditions (WES-RC), which is a survey targeting problems at work [53]. Guided by the WES-RC, a detailed assessment of work barriers, activity limitations, and the participants’ roles and tasks in relation to their work are discussed. Goal setting and problem prioritisation are performed using the Canadian Occupational Performance Measure (COPM), which the OTs are trained to use [54]. Furthermore, the participant’s wish regarding the involvement of relatives and employers is clarified. In addition, the OT registers if the participant receives other rehabilitation offers, such as offers at the job centre, to secure cooperation with relevant partners.

#### Coordinating OT

The coordinating OT is available at agreed-upon telephone hours (also outside the patients’ normal working hours) or by email if there are specific and practical questions that need to be clarified. The coordinating OT can support the participant to establish contact to relevant partners, such as consultants from the municipality, and in navigating the municipality’s offers, etc. Furthermore, the participant is encouraged to involve the employer and the coordinating OT offers meetings with the participant and employer, if accepted by the participant. The coordinating OT also assesses whether there is a need for workplace adjustments or specific aids. In Denmark, this is handled by an OT at the municipal job center, and the coordinating OT will help establish the contact. If needed, a pamphlet called ‘Dear employer, I have arthritis’ is handed out to the participant to give to the employer. The pamphlet was developed at the Danish Center for Expertise in Rheumatology at the DHRD and describes the challenges people with IA may face at work. In addition, the need for individual offers (such as physiotherapy) is coordinated with the participant. The day before each meeting with the coordinating OT, a text message is sent with a reminder to the participant: ‘Dear…I look forward to seeing you tomorrow at…to…’.

The coordinating OT also provides individual support in relation to the goals agreed upon and offers individual support about personal issues related to work. The individual support can encompass concerns, problems with conscience, and negative thoughts. Generally, the focus is on the participants’ self-management and occupational balance, and the coordinator uses elements from Focused Acceptance and Commitment Therapy (FACT) [52]. The coordinating OT and the participant can use up to ten hours throughout the six months VR. The coordinating OT and participant assess the participant’s need together about how much, when, and where the consultations take place. The consultations can take place physically, online, or by phone.

If necessary, a final meeting with relevant partners (e.g., social workers, consultants from the municipality, employers, and relatives) is held to evaluate goals and discuss future plans if the participant needs further rehabilitation or support.

#### Group sessions

The three group sessions run over the first two months of the VR, with one week between each session. The patient can start the group sessions after the initial assessment and goal setting process and when ten participants are recruited. The duration of each session is two hours. There is a focus on supporting the patients to develop relationships with each other and to share experiences.

##### Session 1: Legislative offers

This is presented by a social worker with focus on the general legislative offers for patients with IA, such as compensatory schemes, offers in the municipal job centre, and possibilities for a flexi-job, which is an offer in Denmark in which the municipality pays a subsidy to the employer for citizens who have decreased work abilities and are only able to work less than half time.

##### Session 2: Acceptance of the disease in relation to work

An experienced rheumatology nurse holds this session. The focus is on dealing with lack of understanding at the workplace, gaining information and understanding of the disease, and sharing experiences with the group members.

##### Session 3: Coping strategies

An experienced rheumatology OT holds this session, and the focus is on energy management and balancing work as part of everyday life.

#### Individual consultations

If needed, the patient is offered consultations with different RCs, with a maximum of two consultations per profession. This part of the VR is optional.

##### Social worker

Supports the patient with contacts in the municipality and follows up on whether further support is needed. Discusses specific legislative offers of relevance for the individual participant and the need for job/industry change.

##### Nurse

Disease information and understanding, concerns, and considerations in relation to medical treatment and management of pain, fatigue, and sleep problems.

##### OT: Hand exercises

 Assessment of the need for small aids and bandages. Ergonomic positions in relation to work, sleep, and positioning techniques. Energy management. These consultations can be delegated to other OTs from the coordinating OT.

##### Physiotherapist

 Information about individually tailored physical activity and exercise. Motivation for exercise. Examination and guidance regarding feet and footwear.

##### Referral to a consultant from municipal job centre

 What the job centre offers and the possibility for a home visit or visit at the workplace.

WORK-ON will be feasibility tested in a rheumatology outpatient clinic at the DHRD.

### Feasibility test

According to the MRC framework, the next step is to evaluate WORK-ON in a feasibility test according to fidelity, dose, adaptations, and reach [23]. The feasibility test will include 20 outpatients from the DHRD. Potential participants will be invited through DANBIO, a national rheumatology quality database, if they answer unlikely or not certain to question #6 from the Work Ability Index Questionnaire: ‘Do you believe, according to your present state of health, that you will be able to do your current job two years from now?’ [41, 49].

RCs who are to deliver WORK-ON have received eight hours of training before initiation of the feasibility test; the coordinating OTs received 11 h of training. This included content in WORK-ON, receiving relevant knowledge, facilitation of group sessions and individual consultations, booking and registration of attendance. The coordinating OTs were already trained in using the COPM. Furthermore, the RCs at the DHRD have a person-centred approach and have received training in FACT during four modules each of three hours duration also using elements from the motivational interview as well as the ICF.

As part of the feasibility test, process evaluation is relevant. Interviews of participants and RCs are planned to investigate experiences of mechanisms of impact and contextual factors of importance and to determine the quality of the delivered VR and ideas for subsequent adjustments [55]. If the results from the feasibility study are promising, WORK-ON will be adjusted in order to be able to conduct a larger RCT, in which an evaluation of effectiveness and an economic evaluation can be performed.

The Regional Committees on Health Research Ethics waived the need for formal approval for the feasibility study.

## Discussion

The aim of this study was to describe the process of developing an evidence-based and theoretically based VR, WORK-ON. We consider the MRC framework as suitable in developing WORK-ON, as it includes interacting components.

WORK-ON consists of four components: 1) initial assessment and goal setting, 2) coordinating OTs, 3) group sessions, and 4) individual consultations. Work is often not included in goal setting [40], and we believe that starting with identifying work barriers using WES-RC and goal setting in shared decision making using COPM provides the best opportunities to benefit from participating in WORK-ON. These instruments are considered suitable, as they assess activity problems at work and identify the patients’ occupational balance.

We also found that social support from employers, co-workers, and relatives is an important factor when trying to maintain work. Wilkie et al. have identified similar needs and described that support from the workplace may have a positive effect on the ability to return to work [9]. Interviews with employers, state that they want to be involved in VR to support their employees. This perspective is supported by Jakobsen et al. why we seek to collaborate with employers in the VR, if the patient sees it as relevant [56].

Relevant theoretical approaches were chosen and occupational balance, self-management, self-efficacy, and shared decision making seemed to be the most relevant, based on the participants’ needs and need for support. We chose the three theoretical approaches as they are person-centered, are helping the patients’ balance their everyday life including work as well as supporting them in self-determination. Other relevant theories could have been theories about health literacy and occupational justice [57, 58], but we assessed that the included theories were the most relevant to match the patients' challenges when meeting the labor market.

### Strengths and limitations

We chose to involve different stakeholders: patient research partners, RCs from hospital and municipalities, and researchers in the development process, which we believe has strengthened the development of WORK-ON as this ensured that several perspectives and needs were included. Originally, several workshops were planned as part of the development process. Due to the COVID-19 pandemic, this was changed into individual interviews with RCs to include these perspectives in the development. Although WORK-ON was developed in a Danish health care system, we consider that some elements in WORK-ON may be transferable to other health care systems.

It is a limitation that the employers were not included in the development of WORK-ON. Though, the employers’ participation is planned as a part of the feasibility test if the participant needs it. In this case, the coordinating OT supports this involvement. We did perform interviews with 13 employers to secure the perspectives of the employers.

## Conclusion

We consider WORK-ON as developed successfully based on the MRC framework, as it requires that the programme theory is based on the evidence base. Furthermore, the logic model provides an overview to understand the mechanisms in WORK-ON.

## Supplementary Information

**Additional file 1: Supplementary file 1. **Template of the Intervention and Replication Checklist for WORK-ON

**Additional file 2: Supplementary 2. **Interview guide, rehabilitation clinicians

**Additional file 3: Supplementary file 3. **Interview guide, employers

## Data Availability

The datasets used and/or analysed during the current study are available from the corresponding author on reasonable request.

## Abbreviations

AxSpA: Axial spondyloarthritis
BRAF: Bristol Rheumatoid Arthritis Fatigue questionnaire
COPM: Canadian Occupational Performance Measure
DHRD: Danish Hospital for Rheumatic Diseases
EQ-5D-5L: Health-related quality of life
FACT: Focused Acceptance and Commitment Therapy
IA: Inflammatory arthritis; MRC: Medical Research Council
OBQ: Occupational Balance Questionnaire
OT: Occupational therapist
PROM: Patient reported outcome measures
PsA: Psoriatic arthritis
RA: Rheumatoid arthritis
RCT: Randomised controlled trial
RC: Rehabilitation clinician
VR: Vocational rehabilitation
WAI: Work Ability Index
WES-RC: Work Experience Survey for Patients with Rheumatic Conditions
WPAI: Work Productivity and Activity Impairment questionnaire
