# Supplementary material for: Developing a complex vocational rehabilitation intervention for patients with inflammatory arthritis: the WORK-ON study
Source: BMC Health Serv Res. 2023 Jul 8;23:739. doi: 10.1186/s12913-023-09780-2 (PMC10329797; doi:10.1186/s12913-023-09780-2)
Supplement: Supplementary file 1 — Additional file 1: Supplementary file 1. Template of the Intervention and Replication Checklist for WORK-ON [file 12913_2023_9780_MOESM1_ESM.docx]

**Supplementary file 1.** Template of the Intervention and Replication Checklist for WORK-ON

| Item | Description |
| --- | --- |
| Intervention name | WORK-ON - vocational rehabilitation for persons with inflammatory arthritis. |
| Rationale | Vocational rehabilitation may have an effect on work ability, absenteeism, and job loss in persons with inflammatory arthritis.  Theoretical approaches such as occupational balance, self-management, self-efficacy, and shared decision making underpin WORK-ON.  *Occupational balance* describes an individual’s satisfaction with occupations in their life, the variations between them, and how meaningful they are. Occupational balance is also characterised as the experience of having the right number of occupations to balance time use. In this context, occupational balance involves areas such as paid work, self-care, leisure, and sleep, as well as occupations with different characteristics, including obligatory, voluntary, and paid work (28).  *Self-management* is defined as the ability to manage symptoms, treatments, and the physical and psychosocial consequences from living with a chronic health condition. To be able to manage a chronic health condition, patients have to manage their cognitive, behavioural, and emotional reactions to maintain a satisfactory quality of life. Health professionals must support the patients in making their own decisions, solving their own problems, seeing their own potential, controlling their own situations, and being active in their daily lives (29).  The *self-efficacy* concept relates to beliefs about one’s perceived abilities or inabilities to complete a specific task and not to one’s actual capabilities or performance. This reflects the individual’s subjective assessment of their abilities and skills to successfully achieve their goals (30).  *Shared decision making* is a process and gives opportunities to reflect upon goal setting, wishes, hopes, needs, and dreams. It shifts the power and control between the patient and the clinician and makes the interaction equal. Shared decision making is described as a three-step process: 1) a team talk during which patients and clinicians work together as a team to make decisions regarding care, 2) the option talk during which opportunities are discussed, and 3) the decision talk to make preference-based decisions (31).  *RCs’ skills at the DHRD:*  The ICF is a rehabilitation framework that RCs at the DHRD have as their preconception. The ICF is a framework for organising and documenting information on functioning and disability (51).  RCs have a person-centred approach and are trained in FACT (53), which is a brief intervention for radical behaviour change and motivational interviewing (52), with the latter being a counselling method that involves enhancing a patient’s motivation to change.  Furthermore, the OTs at the DHRD are trained in using the COPM, which is an evidence-based measure for goal setting and prioritising occupations (55). |
| Materials | A detailed manual supports RCs who deliver the intervention in WORK-ON. The manual includes the rationale for and content of WORK-ON and templates for notes in the participant’s electronic health file and epicrisis for the participant’s general practitioner.  The coordinating OT uses the WES-RC, which is a survey targeting challenges at work. Goal setting and prioritisation of occupations are performed using the COPM.  Participants who receive WORK-ON are, if necessary, offered the pamphlet ‘Dear employer. I have arthritis’, which has been developed by the Centre for expertise in arthritis at the DHRD. |
| Training | RCs who are to deliver WORK-ON received eight hours of training before initiation of the feasibility test; the coordinating OTs received 11 hours of training, including information on how to use the WES-RC. Training included content from WORK-ON, relevant knowledge about background information, the theoretical approaches and concepts behind the intervention and VR, facilitation of group sessions and individual consultations, and booking and registration of attendance. Furthermore, the RCs at the DHRD had previously received training in FACT across four modules, each lasting three hours in duration. |
| Coordinating OT | *Initial assessment and goal setting:*  A two-hour physical meeting with an initial assessment and goal setting process is performed by a coordinating OT who has experience with the rehabilitation of patients with IA and the challenges they face in the labour market. The coordinating OT starts with a structured interview guided by the WES-RC, which is a survey targeting problems at work. Guided by the WES-RC, a detailed assessment of work barriers, activity limitations, and the participant’s roles and tasks in relation to their work are discussed. Goal setting and prioritising occupations are performed as part of using the COPM, which the OTs are trained to use. Furthermore, the involvement of relatives and employers is discussed, particularly the extent to which this involvement is wanted by the participant. The OT registers other rehabilitation offers that the participant is already receiving, such as offers at the job centre, to secure cooperation with relevant partners.  *Coordinating OT:*  The coordinating OT is available at agreed-upon telephone hours (outside the participant’s normal working hours) or by email if there are specific and practical questions that need to be clarified. The coordinating OT can support the participant in establishing contact with relevant partners, such as consultants from the municipality, and in navigating the municipality’s offers and so forth. In addition, the need for individual offers (e.g., for physiotherapy) is coordinated with the participant. The day before each meeting with the coordinating OT, a text message is sent with a reminder to the participant: ‘Dear…I look forward to seeing you tomorrow at…to…’.  The coordinating OT also provides individual support in relation to the goals agreed upon and offers individual support regarding personal issues related to work. The individual support can encompass concerns, problems with conscience, and negative thoughts.  The coordinating OT and the participant can use up to ten hours during the six months of VR. The coordinating OT and participant work out how much, when, and where the consultations take place. The consultations can take place physically, online, or by phone.  If necessary, a final meeting with relevant partners (e.g., social workers, consultants from the municipality, employers, and relatives) is planned to evaluate goals and discuss future plans in case the participant needs further rehabilitation or support.  The participants can receive up to seven consultations with the coordinating OT throughout the intervention. |
| Group sessions | The participants all attend three group sessions during the first two months of the VR, with one week between each session. The participants start at the group sessions after the initial assessment and goal-setting process. The duration of each session is two hours. There is a focus on supporting the participants to develop relationships with each other and share experiences. In each group there are 6 -10 participants.  *Session 1:*  Legislative offers. A social worker presents the general legislative offers for people with IA, such as compensatory schemes, offers in the municipal job centre, and possibilities for a flexi-job, which is an offer in Denmark through which a municipality subsidises an employer for citizens who have decreased work ability and thus are only able to work less than half time.  *Session 2:*  Acceptance of the disease in relation to work. An experienced rheumatology nurse focuses on dealing with lack of understanding at the workplace, gaining information and understanding of the disease, and exchange of experiences among the participants.  *Session 3:*  Coping strategies. An experienced rheumatology OT focuses on energy management and balancing work as part of everyday life. |
| Individual consultations | If needed, individual consultations with the relevant RCs (the social worker, nurse, OT, and physiotherapist) are offered after the group sessions.  *Social worker:*  Supports the participant with contacts in the municipality and follows up on whether further support is needed. Discusses the specific legislative offers of relevance for the individual participant and, if needed, opportunities for job/industry change.  *Nurse:*  Addresses disease information and understanding, concerns, and considerations in relation to medical treatment and management of pain, fatigue, and sleep problems.  *OT:*  Facilitates hand exercises. Assesses the need for small aids and bandages. Recommends ergonomic positions in relation to work, sleep, and positioning techniques. Supports energy management. These consultations can be delegated to other OTs besides than the coordinating OT.  *Physiotherapist:*  Provides information about individually tailored physical activities and exercises. Supports with motivation for exercise. Examines and gives guidance regarding feet and footwear.  *Consultant from municipal job centre in Denmark:*  The consultant’s purpose is to discuss the participant’s needs in relation to what the job centre can offer and the challenges the participant faces at work. This consultation takes place at the municipal job centre, in the participant’s home, or at the workplace and may include the following considerations:   - What does the job centre offer? - Possibility of a home visit - Assessment of the need for aids - Possibility of a workplace visit   - Is there an OT associated with the workplace who can be involved?   - If relevant, the employer is involved and an agreement is made about whether a workplace visit is possible   - Information about how the participant and employer can apply to the municipality for a workplace assessment - Information about how anything done in relation to the participant’s ability to work must be written down in a personal file for use in a possible later assessment for a flexi-job - Assistive device assessment |
| Delivery | WORK-ON is delivered at the DHRD as an outpatient intervention. It is possible to deliver some of the consultations by phone or online (using Zoom). All RCs are experienced in rheumatology rehabilitation. |
| Duration and dose | WORK-ON runs over six months with 9–18 consultations with the coordinating OT and other RCs including group sessions. There are three group sessions of 2,5 hours each (including dinner). Each participant can receive 2 hours of consultation(s) per RC. The maximum number of hours for the participants is 28 hours. |

Abbreviations: Canadian Occupational Performance Measure (COPM), Danish Hospital for Rheumatic Diseases (DHRD), focused acceptance and commitment therapy (FACT), inflammatory arthritis (IA), the International Classification of Functioning, Disability and Health (ICF), occupational therapist (OT), rehabilitation clinician (RC), Work Experience Survey for Patients with Rheumatic Conditions (WES-RC).
