# Supplementary material for: Developing a complex vocational rehabilitation intervention for patients with inflammatory arthritis: the WORK-ON study
Source: BMC Health Serv Res. 2023 Jul 8;23:739. doi: 10.1186/s12913-023-09780-2 (PMC10329797; doi:10.1186/s12913-023-09780-2)
Supplement: Supplementary file 2 — Additional file 2: Supplementary 2. Interview guide, rehabilitation clinicians [file 12913_2023_9780_MOESM2_ESM.docx]

***Supplementary 2: Interview guide, rehabilitation clinicians***

| **Theme** | **Questions** | **Supplemental questions** |
| --- | --- | --- |
| **Background questions** | What is your profession?  When did you graduate?  What are your job functions?  What are your primary work tasks?   - When do normally you encounter the citizens/patients?   What experiences do you have with work-related issues?   - Do you ask how the patient is coping with his/her ability to master their work?   How do you experience the cooperation between the municipality and the hospital in relation to the patients’ work-related problems? | What does a typical workday look like for you?  What theory/evidence do you use in your work?  Do you use special work methods/work tools or research tools?   - How do they work?   How are your professional skills compared to your colleagues’?   - What does your subject group contribute in particular?   How do you consider interdisciplinary collaboration in your work? |
| **Experience** | Which challenges do you experience that people with IA face in their work?  How do you measure what the patient’s challenges are?   - Can you give an example (preferably several)? What did you/the interdisciplinary team do? - Was there any cooperation with the municipality/hospital? - Is it possible for you to explore this thoroughly enough? - What does it take? - What suggestions do you have so that there can be a greater focus on this challenge? - How do you think we can best help citizens/patients with work-related issues and problems?   Do you advise/guide the citizens/patients in their possibilities in the labour market?   - If not, what is the reason? Time, knowledge, other?   Some of the patients say that they have a scepticism towards the ‘system’.   - Is this also something you experience? - What significance do you think this has for their work-related intervention? | E.g., fatigue, pain, economy, physical limitations  E.g., personal assistance, flex job, application for assistive technology, etc. |
| **Balance and values** | Citizens/patients describe having a job as very important.   - How do you perceive that citizens with arthritis feel? - Does it matter to the process what value the citizens have about going to work?   Citizens/patients report that it is difficult to balance their energy between work and family life and that work takes most of their energy.   - What opportunities do you have to support how the citizens/patients best manage their energy throughout the day? | Do you use other strategies/methods? |
| **Vocational rehabilitation offer** | What are your best experiences with vocational rehabilitation?  With your knowledge and experience, what would the best vocational rehabilitation offer look like?   - How should you assess the citizen’s/patient’s challenges and need for support? - What would need to be included in vocational rehabilitation?   What special knowledge from your own profession do you include in your work?  Are there interventions missing for people with inflammatory arthritis to better keep their jobs?  Do you have any suggestions about how the cooperation between the municipality and the hospital can be improved in this area? |  |
| **Relations** | Some of the patients do not tell their employer about their arthritis.   - Do you recognise it? - What significance does it have for the patient?   Many of the patients find that family and colleagues do not understand them and thus cannot provide the support that is needed.  Do you involve close relations/relatives in the rehabilitation process?   - How do you do it? - If so, to what extent?   Do you have the opportunity to involve the patient’s workplace (employer, colleagues)?   - How?   Do you collaborate with other departments around the patient?   - E.g., job centre, health centre, hospital, case managers, etc. | What do you do if a patient tells you that he or she has not told their employer about the rheumatic disease?  If not, do you wish you had the opportunity? |
