# Supplementary material for: Developing a complex vocational rehabilitation intervention for patients with inflammatory arthritis: the WORK-ON study
Source: BMC Health Serv Res. 2023 Jul 8;23:739. doi: 10.1186/s12913-023-09780-2 (PMC10329797; doi:10.1186/s12913-023-09780-2)
Supplement: Supplementary file 3 — Additional file 3: Supplementary file 3. Interview guide, employers [file 12913_2023_9780_MOESM3_ESM.docx]

***Supplementary file 3: Interview guide, employers***

| **Theme** | **Questions** | **Supplemental questions** |
| --- | --- | --- |
| **Background questions** | What is your profession?  When did you graduate?  What are your job functions?   - For how long have you been working? - Is there an HR function at the workplace? - Type of company? - Company size? |  |
| **The employer’s experience and role in relation to employees with a rheumatic disease** | What are your experiences with job retention and employees with arthritis?  Can you briefly describe the company’s policy in connection with sick leave?  As part of your job function, are you responsible for conversations with employees on sick leave?   - Who typically participates in these conversations?   Do you currently know an employee who has a rheumatic disease?   - If not, when was the last time you had an employee   with a rheumatic disease?  Did you experience special challenges for the employee who has a rheumatic disease?  Do you feel that the employee has been open about his/her disease?   - If not, what challenges does it pose for you as an employer?   What thoughts do you have about the company’s finances in connection with an employee’s illness?  Do you make special socio-economic considerations when you, as a company, handle sick leave? | Can you give an example of how a conversation takes place with an employee who is at risk of sick leave or has to return after sick leave?  Sick leave, adjustment in work tasks, workplace adjustments   - What special needs did the employee have? - What did you specifically implement in relation to sick leave? |
| **The contact for the job centre**  *Legislative framework* | What do you need as an employer in connection with hiring/retaining an employee with arthritis?  What type of information do you need in relation to prevent job loss at an employee with arthritis?  How would you describe your cooperation with the job centre regarding the employee with arthritis?   - Do you have a permanent contact person at the job centre? - Do you feel that the job centre has necessary and useful knowledge about citizens with arthritis? - Would you like to be involved more?   Can you describe a situation in which an employee has returned after a long illness?  How would you describe your knowledge of the sickness allowance law?  Did you miss anything in the employee cases you have already been involved in?  Do you know about the fast track scheme, and if so, what are your experiences with the scheme?  Do you know about the disability compensation schemes?  What is your knowledge of the various flexible job opportunities?   - Could you imagine using one of the schemes if an employee had a permanently reduced ability to work due to arthritis? | Has anything been particularly challenging or positive?  E.g., sick leave reimbursement, termination of sick leave, §56 agreement, extension options, partial sick leave, retention plans  Assistive devices, personal assistance, priority access  Flex jobs, mini flex jobs (less than 10 hours) and retention flex jobs |
| **Occupational justice**  **Occupational balance**  *The right to develop*  *The right to autonomy*  *The right to experience activities as meaningful and value-creating* | Do you experience that the employee has difficulties with balancing work and everyday life?   - Do you have the impression that the employee is open about this with you?   What do you think about the necessity for there to be a balance between the employee’s everyday life and working life?  Do you find that there is anything else other than the disease that has an impact on whether the employee with arthritis can perform or stay at work?    Is it possible to offer meaningful work tasks at your workplace while, at the same time, taking patients’ needs into account?  Is it possible for the employee with arthritis to have an influence on the work tasks?  Do you feel that the employee with arthritis has the resources to think about his or her own professional development?  Do you feel that there have been changes in the ways in which the employee is included in the social and collegial environment?  Have there been disagreements about which tasks the employee with arthritis had to perform?  Have you experienced legislation and/or the job centre resisting the employee’s wishes in relation to employment/retention?  Has the employee at any time expressed that they feel isolated or can no longer recognise themselves (identity)?  Do you feel that you can offer the employee with arthritis the same opportunities as employees without illness and limitations in working ability? | Introduction to the concepts  Has it been necessary/possible to relocate the employee to another department? |
